# Outcome measures assisting treatment optimization in multiple sclerosis

**DOI:** 10.1007/s00415-021-10674-8

**Published:** 2021-08-02

**Authors:** Gabriel Pardo, Samantha Coates, Darin T. Okuda

**Affiliations:** 1 OMRF Multiple Sclerosis Center of Excellence, Oklahoma Medical Research Foundation, 820 NE 15th Street, Oklahoma City, OK 73104 USA; 2 Excel Medical Affairs, Horsham, UK; 3 Department of Neurology, University of Texas Southwestern, Dallas, TX USA

**Keywords:** Multiple sclerosis, Monitoring modalities, Function, Dexterity, Cognition

## Abstract

**Objective:**

To review instruments used to assess disease stability or progression in persons with multiple sclerosis (pwMS) that can guide clinicians in optimizing therapy.

**Methods:**

A non-systematic review of scientific literature was undertaken to explore modalities of monitoring symptoms and the disease evolution of MS.

**Results:**

Multiple outcome measures, or tools, have been developed for use in MS research as well as for the clinical management of pwMS. Beginning with the Expanded Disability Status Scale, introduced in 1983, clinicians and researchers have developed monitoring modalities to assess all aspects of MS and the neurological impairment it causes.

**Conclusions:**

Much progress has been made in recent decades for the management of MS and for the evaluation of disease progression. New technology, such as wearable sensors, will provide new opportunities to better understand changes in function, dexterity, and cognition. Essential work over the decades since EDSS was introduced continues to improve our ability to treat this debilitating disease.

## Introduction

Multiple sclerosis (MS) is an inflammatory neurologic disease with a varied presentation, and diagnosis is made clinically [1–4]. Once diagnosed, the type and speed of symptom progression in MS vary, making the clinician’s job of assessing disease evolution and treatment responses a perpetual challenge.

Multiple outcome measures, or tools, have been developed for use in MS research and clinical management of persons with MS (pwMS). Such tools have been designed to help determine the progression and severity of disease, including inflammatory activity [clinical relapses or new magnetic resonance imaging (MRI) lesions] and neurodegeneration (progression in absence of relapses). These tools are also used to identify evidence of a response to treatment. To ensure effective use, the practicing clinician must first gain an understanding of the benefits and downsides of each tool to determine whether to incorporate it into a patient’s evaluation and therapeutic decisions. If the tool is to be incorporated, the clinician must then consider how to effectively implement it and interpret the results. Currently, only a few of the tools in existence are commonly used in research and patient management [5].

Here we provide an overview of tools that can be used to evaluate the functional (Table 1) and neuroanatomical (Table 2) components of MS, highlighting new data on potential MS biomarkers and how they may be utilized by clinicians in the future. Some patient-reported outcome tools are presented in Table 3 for reference, but they are not detailed in this review. Our aim is to enable clinicians to more accurately assess stability or progression in pwMS and to guide treatment optimization, even in subclinical progression.

**Table 1:** Commonly used tools for the assessment of functional change in individuals with multiple sclerosis

Test	Function tested	Description	Equipment	Process	Utility	Threshold for clinically meaningful change
T25FW [19]	Walking disability	Measures time (in seconds) to walk 25 feet	$\bullet$ Unobstructed open space measuring 25 feet (no turns permitted) $\bullet$ Timer (e.g., stopwatch) $\bullet$ Mechanism for recording scores (e.g., pen and paper) $\bullet$ Assistive walking devices (optional)	$\bullet$ Ask individual to walk the distance as quickly and safely as possible, stopping beyond the finish point to avoid a reduction in speed $\bullet$ Repeat once	$\bullet$ Routine management and clinical trials	$\bullet$ 20% change [19, 23, 24]
6MWT [30]	Gait	Measures distance walked (in meters) in 6 min	$\bullet$ Unobstructed open space (turns permitted) $\bullet$ Assistive walking devices may be used $\bullet$ Timer (e.g., stopwatch) $\bullet$ Mechanism for recording score (e.g., pen and paper) $\bullet$ Assistive walking devices (optional)	$\bullet$ Ask individual to walk for 6 min at maximal speed	$\bullet$ Clinical practice and clinical trials	$\bullet$ 20% change [22]
TUG [39]	Basic mobility	Measures the time taken (in seconds) to stand from a chair, walk a fixed distance, return, and sit down	$\bullet$ 2-armed chair positioned in a space that allows a 10-foot walk $\bullet$ Assistive walking devices may be used $\bullet$ Timer (e.g., stopwatch) $\bullet$ Mechanism for recording score (e.g., pen and paper) $\bullet$ Assistive walking devices (optional)	$\bullet$ Ask individual to rise from the chair, walk to a mark 10 feet away, turn, and return to a seated position $\bullet$ Repeat (once or twice)	$\bullet$ Clinical practice to monitor effects of medical and rehabilitation treatment	$\bullet$ Improvement of 23–24% and deterioration of 30–31% [40]
9-HPT [50]	Dexterity (upper extremity [hand/arm] funsction)	Measures time taken (in seconds) or speed (in pegs/second) to complete the task	$\bullet$ Table and chair $\bullet$ Shallow container with 9 pegs and a block containing 9 holes for the pegs to fit in $\bullet$ Timer (e.g., stopwatch)	$\bullet$ Ask the individual to sit and, using 1 hand, put pegs 1 at a time into the holes as quickly as possible; once completed, ask the individual to remove the pegs (with the same hand) 1 at a time and place back in the container $\bullet$ Repeat once with the same hand $\bullet$ Repeat twice with the other hand	$\bullet$ Clinical practice and self-assessment, and as part of the MSFC in clinical trials	$\bullet$ 20% change [133]
SDMT [65]	Cognition (processing speed)	Measures time taken (in minutes/seconds) to complete the task	$\bullet$ Set of symbols and numbers $\bullet$ Timer (e.g., stopwatch) $\bullet$ Mechanism for recording score (e.g., pen and paper) $\bullet$ Symbol test, including mechanism for recording response (electronic versions of the SDMT may be available)	$\bullet$ Give the individual a set of symbols corresponding to a series of numbers $\bullet$ Ask the individual to write down the symbols corresponding to the series of numbers	$\bullet$ Clinical practice and trials assessing cognitive processing speed	$\bullet$ 4-point score change, 10% reduction in score, or change in 0.5 standard deviations [63]
LCLA [76]	Visual disability	Measures impaired low-contrast vision at multiple letter sizes	$\bullet$ Sloan Eye chart $\bullet$ Retro-illuminated cabinet or darkened room $\bullet$ Mechanism for recording score (e.g., pen and paper)	$\bullet$ Individual seated 2 m from chart $\bullet$ Individuals read letters aloud top to bottom and left to right $\bullet$ Scores are quantified as the number of letters identified on a Sloan eye chart (maximum 70)	$\bullet$ Clinical trials	$\bullet$ 7-point change [76]

*6MWT* 6-Minute Walk Test, *9-HPT* 9-Hole Peg Test, *LCLA* low-contrast letter acuity, *SDMT* Symbol Digit Modalities Test, *T25FW* Timed 25-Foot Walk, *TUG* Timed Up and Go

**Table 2:** Commonly used tools for the assessment of neuroanatomical change in individuals with multiple sclerosis

Test	Function tested	Utility	Interpreting results
MRI [81]	MS disease activity in the brain	$\bullet$ Routine clinical practice (diagnosis, monitoring disease progression, prognosis); primary or secondary endpoint in intervention trials[82, 87]	$\bullet$ New MRI lesion formation indicates active inflammation/active disease in relapsing MS and may indicate poor treatment outcome $\bullet$ Presence of gadolinium-enhancing and spinal cord lesions at diagnosis predict long-term development of secondary progressive MS and physical disability and may influence initial treatment selection
OCT [100]	Neurodegenerative changes in the retina	$\bullet$ Routine clinical practice and clinical trials	$\bullet$ Thinning of retinal nerve fiber layer indicates MS disease progression [99, 101]

*MRI* magnetic resonance imaging, *MS* multiple sclerosis, *OCT* optical coherence tomography

**Table 3:** Commonly used patient-reported outcome tools

Test	Function tested	Summary
MSQOL-54 [134]	Patient-reported quality of life	$\bullet$ Based on SF-36 with additional MS-specific items
MFIS [135, 136]	Fatigue	$\bullet$ Assessment of patient fatigue in terms of physical, cognitive, and psychosocial function
SF-36 [137, 138]	Patient-reported quality of life	$\bullet$ Generic life questionnaire with limited utility for MS parameters aside from cognitive function

*MSQOL-54* 54-item Multiple Sclerosis Quality of Life questionnaire; *MFIS* Modified Fatigue Impact Scale, *MS* multiple sclerosis, *SF-36* 36-item Short Form Health Survey

The different instruments are presented in three categories: *functional*, describing evaluations of motor, ambulation, and cognitive performance; *anatomical*, reviewing imaging of the brain, spinal cord and retina; and *biological*, addressing the evolving area of biomarkers.

## Methods

A non-systematic review of scientific literature was undertaken to explore modalities of monitoring symptoms and the disease evolution of MS. We searched PubMed in Jan-Feb 2020 using the following terms and limiting to English language and humans and papers since January 2000: “multiple sclerosis” and “Expanded Disability Status Score”, “Timed 25-Foot Walk”, “Six-Minute Walk Test”, “Timed Up and Go”, “9-Hole Peg Test”, “Symbol Digit Modalities Test”, “Low-contrast letter acuity”, “magnetic resonance imaging”, Optical Coherence Tomography”, “biomarkers”, and “neurofilament”. A similar procedure was followed in April–May 2021 to include “Multiple Sclerosis Functional Composite” and “Paced Auditory Serial Addition Test”. A manual search of papers included was also done to identify other possible references, including some that were relevant from the period before January 2000.

### Functional instruments

#### Expanded Disability Status Score (EDSS)

The EDSS was introduced in 1983 to quantify neurological impairment in pwMS [6]. It is used to score patients across eight functional groupings on a step scale of 0–10. The disability scoring can be simplified to mild [≤ 4.5; able to walk without any aid (considered fully ambulatory)], moderate [5–6.5; ranging from ambulatory without aid or rest for ~ 200 m to requiring constant bilateral assistance (canes, crutches, or braces) for walking ~ 20 m without resting], and severe (7–10; ranging from being unable to walk beyond ~ 5 m even with aid, to death) [6]. Natural history studies based on EDSS have shown an accelerated phase of progression beginning around a score of 4.0 [7–9]. In an MS population treated at a clinic in Ontario, Canada, Weinshenker et al. observed that patients spent the shortest mean times at EDSS 4 and 5 (1.22 and 1.25 years, respectively) than at any other EDSS score [9].

Since its introduction, EDSS has been a standard instrument for assessing patients with MS and charting status changes. It is widely used in clinical trials to assess the effectiveness of clinical interventions and in the routine clinical assessment of disease progression in pwMS [10]. A second assessment of EDSS change is generally done at a minimum of 3 months to confirm that the progression was not temporary for trials of 2- to 3-year duration [11]. Moreover, confirmed persistence of progression at 3 months accurately estimates irreversible progression in 70% cases at 5 years (i.e., may result in the identification of temporary disability changes in 30%). More accurate evaluation of irreversible disability is seen when extending the confirmation periods (6 months, 74%; 12 months, 80%, 24 months, 89%) [12]. Limitations such as low sensitivity to change and underrepresentation of fatigue, visual function, and cognitive impairment, however, have been noted and discussed [13]. The functional groupings of EDSS are largely contingent on non-linear loss of ambulatory ability and do not include scoring for loss of cognition or other neurological impairments. Although EDSS retains its place in the language of MS assessment, numerous instruments and tests have been proposed and validated to fill patient monitoring gaps.

#### Multiple sclerosis functional composite (MSFC)

The multiple sclerosis functional composite (MSFC) is a multidimensional, three-component scale to assess the degree of functional impairment in MS patients. It was developed by the National MS Society (NMSS) in 1994 to address the limitations and unidimensionality of other functional status outcomes [10, 14]. After a rigorous analysis of candidate outcome measures, the following tests were included: Timed 25-Foot Walk (T25W) for leg function and ambulation, 9-Hole Peg Test (9HPT) for arm and hand function, and Paced Auditory Serial Addition Test (PASAT) for cognitive function, all of which are described separately in this publication. An integrated MSFC score is calculated using z scores from the three components. The entire composite measure takes approximately 20 min to complete [15].

The primary goal for creating the MSFC was to develop a new clinical outcome measure for use in MS clinical trials [15], and it has proven a useful outcome in Phase 3 trials of disease-modifying agents for MS as both a primary and secondary outcome measure [10].

There has been robust support for the validity of the MSFC, with studies showing correlation with disability as measured by the EDSS, disease course and patient self-report measures of symptoms and QoL. Some studies have also shown better correlation between MSFC and MRI measures of cerebral lesion burden and atrophy than seen with the EDSS, but this correlation is inconsistent [10, 16].

A systematic literature review evaluating the validity of the MSFC compared with the EDSS found that while the EDSS has some documented weaknesses in reliability and sensitivity to change, the MSFC is limited in its learning effects of the PASAT, the z score method used to calculate the total score, low acceptance among patients and lack of a visual dimension [10]. Both tools are suitable for detecting the effectiveness of clinical interventions and to monitor disease progression. Of the two measures, EDSS appears to be the more widely used in clinical trials and its international acceptance facilitates comparison of data between studies [10]. Despite some limitations, both instruments are accepted as endpoints although MSFC is often used as a secondary parameter [10].

### Gait

#### Timed 25-foot walk (T25FW)

The T25FW has been used to measure gait speed in pwMS for > 3 decades in both clinical and research settings [17]. It was initially part of the Ambulatory Index [18], supporting MS research and clinical practice, and was subsequently incorporated into the MSFC for use in clinical trials [8]. The T25FW has been used to assess interventions in drug and rehabilitation trials and is useful to assess ambulation changes in the clinical setting [19]. The T25FW is conducted using a premeasured, linear, unobstructed, 25-foot distance. From an initial standing position, the individual is instructed to safely walk the measured distance as quickly as possible, going past the end measurement to avoid slowing down at the end. A second measurement follows. Use of assistive devices to accomplish the task is permitted. The time (in seconds) to complete each segment is recorded and then averaged to obtain a score. Speed can also be calculated in feet per second [19].

A recent meta-analysis of T25FW studies identified 50 articles that included 6303 individuals with MS and 1377 healthy controls, providing evidence for the utility of the T25FW as a gait assessment in MS [20]. Individuals with MS were 55% slower in the T25FW than healthy controls (mean difference − 2.4 s), with an effect size of − 0.92. Performance on the T25FW was worse in those with greater impairment as individuals with mild MS were 51% faster than those of individuals with moderate to severe MS (mean difference − 5.5 s), with an effect size of − 1.02. In addition, performance on the T25FW was worse in individuals with progressive MS compared with those who had a relapsing clinical course. Those with a relapsing course had a 67% faster completion on the T25FW (mean difference − 13.4 s), with an effect size of − 1.36. All of these effect sizes are indicative of clinically meaningful differences [20].

Standardized scoring of the T25FW calculates a *z* score [8]. Because this scoring system is challenging to understand and implement in clinical practice [21], alternative methods of interpreting meaningful change have been suggested. For instance, a minimum detectable change of 2.7 s in T25FW time has been described [22]. A time of 6 to 7.99 s correlates with meaningful life changes due to disability, whereas a time of ≥ 8 s is associated with a permanent disability, use of a walker, and inability to perform daily tasks [17]. However, an approximately 20% change in the time needed to complete the T25FW has most often been described as a meaningful change [19, 23, 24] and was used in MS clinical studies of dalfampridine for the improvement of walking speed [24–26]. Minimum detectable changes of 21–36% have been calculated in some studies, with the variation explained by differences in MS severity [28, 27].

The T25FW correlates well with EDSS (Spearman coefficient 0.56; 95% CI 0.55–0.58) [21]. Nevertheless, some limitations have been suggested. For instance, directions provided must be clear and consistent in order to have the best evaluation of the individual’s speed [8]. In addition, scores on the T25FW separated by 1 week have been observed to be consistently faster the second week, indicating a practice effect [29]. Researchers have also noted a floor effect, by which results in patients with less disability are similar to those of healthy controls [21]. Also, as the T25FW is solely a measure of speed, gait quality is not captured and clinicians need other measures to evaluate fall risk, endurance, and balance. Indeed, some recognize that the T25FW is particularly effective as part of a group of evaluations in MS rather than a standalone test [21].

#### Six-minute walk test (6MWT)

The 6MWT is a measure of motor fatigue validated in 1982 as a quicker alternative to the 12-min walk test for evaluating pulmonary function [30]. It was validated for MS in 2008 [30] and since then has been broadly incorporated into clinical practice and has more recently been used as a primary outcome measure in clinical trials of interventions aimed at improving gait in MS [31]. For MS, general modifications made to the original American Thoracic Society guidelines include suggestions for rest during testing (participant may lean against a wall) and standardization of language for encouragement from evaluators [30, 32]. Since performance on 6MWT is influenced by pulmonary function [32], it is may be preferable to consider it as a measure of walking endurance rather than a true measure of motor fatigue.

The 6MWT includes a measured course, either continuous or with a defined turning point, that is indoors, flat, and without obstacles. The participant walks at a maximum safe speed for 6 min, and the distance traveled is recorded. An examiner may walk behind the individual with a measuring wheel without setting a pace, and participants may use their current walking assistance device if it is regularly used [30, 32]. When validated in MS patients, to maximize effort and better assess motor fatigue, the script for the 6MWT was modified from that used in patients with pulmonary orders; namely, by eliminating instructions for permitted rest during testing, emphasizing speed and excluding encouragement phrases. Modified 6 MW instructions were read prior to each walk. Subjects used their typical assistive device and walked back and forth in a 175-foot hallway, pivoting at each end of the hall. The floor was marked in 8.5-foot increments. Distances walked during each minute and total distance were recorded [30].

Measurement of the first minute of the 6MWT compared to the final minute has been described as a way of identifying motor fatigue, with a 15% decrease in distance during the first minute to distance during the final minute indicating motor fatigue [33]. The minimum detectable change in the 6MWT has been reported to be 88 m, and a 20% change from one measurement to the next is clinically relevant [22].

A meta-analysis of studies employing the 6MWT identified 34 articles with results from 2683 pwMS and 521 healthy controls, confirming the utility of the 6MWT as a measure of endurance in MS [34]. On average, pwMS walked 177.92 m less than healthy controls, for a mean effect size of − 1.87 [standard deviation (SD) 0.17; *p* < 0.001]; pwMS with mild disability walked 185.19 m farther than pwMS with moderate to severe disability, for a mean effect size of 1.83 (SD 0.10; *p* < 0.001). Moderators of response were evident. The design of the course, continuous versus straight with 180º turns at either end, impacted the effect size, with larger effects between individuals with or without MS and mild or moderate to severe disability noted when a continuous course was used. In addition, a larger effect size was noted between pwMS and healthy controls when encouragement/feedback was provided [34].

Results from the 6MWT have been shown to correlate with results on the T25FW [35, 36], and correspondence with EDSS scales has been reported. Using a convenience sample, European researchers demonstrated that after physical rehabilitation, individuals with MS and an EDSS score ≤ 6.5 had better changes in scores with the 6MWT than with the T25FW (0.64 vs 0.59) [37]. In addition, individuals identified as having moderate to severe disability (EDSS 4.5–6.5) rather than mild disability (EDSS ≤ 4) showed superior responsiveness in the 6MWT compared with the T25FW (0.62 vs 0.57). Hence, longer walking tests such as the 6MWT may be a more sensitive measure than the T25FW in detecting improvements in walking after physical rehabilitation in patients with mild and moderate-severe levels of disability.

### Balance

#### Timed Up and Go (TUG)

Balance is impaired in pwMS, and impairment can be more severe than it is in individuals with other conditions such as Parkinson’s disease [38]. TUG is a measure of balance originally designed in 1986 for the frail elderly [39]. The test used primarily to monitor the effects of treatment in clinical practice[35] is performed beginning with the individual in a seated position in a two-armed chair. The individual is instructed to rise from the chair, walk to a mark that is 10 feet (3 m) from the chair, turn, and return to a seated position in the chair. Time is measured in seconds from the initial seated position to the return to sitting. The individual should use any walking aid that they require in daily life and wear their regular footwear, but no assistance is allowed during the test [39]. The test may be repeated and the average time recorded. Some data suggest that a single attempt is sufficient for evaluation [40], while other data support the averaging of two consecutive measures [41].

TUG evaluates multiple aspects of daily living functionality: standing up, sitting down, and turning, in addition to walking speed. Test–retest reliability and reproducibility have been confirmed [35, 42], and TUG has been shown to be reliable and responsive with no detected learning effect [41]. TUG significantly correlates with EDSS (score 2.0–6.5 and no relapse within 30 days) and T25FW in individuals with MS and is a stronger predictor of EDSS score than the T25FW [35]. TUG also strongly correlates with other measures of functionality, disability, and ambulatory mobility in pwMS, and significantly correlates with balance and self-reported balance confidence [43]. TUG times strongly correlate with 6MWT times [44] and with balance measurements among individuals with MS and low-minimal disability [45]. In adults with mild MS (EDSS ≤ 4) at two university hospital outpatient centers, the mean TUG test time was 7.7 (range 5.0–12.5; SD 1.7) seconds [41]. Time to completion for females was 32% longer than for males (time difference 1.9 s, *p* < 0.05). The minimum detectable change reported for TUG was 10.6 s [40].

Although a study of the Khuzestan MS Patients' Society (Iran) demonstrated that TUG test scores were predictive of falls in individuals with MS [46], other MS studies show that TUG is unable to discriminate between those with and without a fall history [47–49].

### Dexterity

#### 9-Hole Peg Test (9-HPT)

Impaired function of the upper extremities is a common consequence of MS [50, 51]. The 9-HPT is an evidence-based, standardized, quantitative test of hand and arm function that was first published in 1971 [52, 53] and was later incorporated into the MSFC [8]. To perform the timed test, an individual is instructed to use one hand to insert nine pegs into a block with nine holes [52]. Once the pegs are in the holes, the individual removes them, one at a time, and places them in a container. The score can be recorded as time taken or speed (pegs per second) for dominant and non-dominant hands individually [50].

The 9-HPT has high inter- and intra-rater reliability [54]. In 69 individuals with MS, intra-class correlation coefficient values for test–retest reliability over 1 week ranged from 0.902 to 0.972, exceeding the threshold for strong reliability (intra-class correlation coefficient > 0.80) [50]. However, performance on the 9-HPT may be sensitive to practice effects, and three or four administrations should be given prior to a baseline assessment if accurate assessments of change over time are needed [16, 54]. The majority of improvement on 9-HPT occurs within the first 2 months following a clinical MS relapse, but improvements have been observed for up to 12 months following a relapse [55].

Increases in 9-HPT score are associated with long-term MS-related disability [21, 56]. A 20% increase in 9-HPT score indicates a clinical impact [53], with changes in 9-HPT associated with diverse functional domains on Guy’s Neurological Disability Scale, including sexual, mood, upper- and lower-limb disabilities, and fatigue [56]. In a study involving 105 people with MS treated with slow-release fampridine, minimal clinically important difference for 9-HPT from pre- to post-treatment was 3.0 s (or 10.7% [range 0.0–15.3%]) [57]. Minimal detectable change for the 9-HPT is smaller for speed measures than for time measures in the non-dominant hand (20.5% and 29.1%), dominant hand (18.6% and 19.4%), and globally (mean of both hands; 12.2% and 15.9%) [50].

The 9-HPT may be particularly sensitive in detecting clinical changes in individuals with progressive MS [58]. A cohort study conducted among such individuals revealed that early changes in 9-HPT score (identified over an initial 1–2 years) were significantly associated with walking limitations ≥ 5 years later [59]. In patients with MS, changes in 9-HPT score have been linked with grey matter damage in the cerebellum, frontal cortex (specifically, Brodmann area 44), and spinal cord; and with damage to white matter in brain areas such as the corpus callosum, cerebral peduncles, internal capsule, and posterior thalamic radiations [60–62].

### Cognition

#### Symbol Digit Modalities Test (SDMT)

Changes in cognitive function are commonly observed in pwMS at any age; prevalence ranges from 34 to 65% in adults and is approximately 33% in individuals aged < 18 years [63]. Cognitive impairment, typically in the form of reduced information-processing speed, occurs in all MS phenotypes and may anticipate progression/conversion to secondary progressive MS or more severe disability (EDSS 4.0) [63, 64]. Identifying these deficits in their onset can support early therapeutic intervention [63]. Indeed, cognitive impairment at initial diagnosis predicts disability progression and conversion to secondary progressive MS [63, 64].

The SDMT takes about 5 min to administer. The subject receives a reference key and has 90 s to pair specific numbers with given geometric figures, being scored on accuracy. Scores are not subject to interpretation by the test administrator. Results are minimally affected by the individual’s age, sex, and educational status; and the test shows only modest practice effects [21, 65]. In addition, the SDMT shows no evidence of skewing, or floor or ceiling effects [21]. The SDMT is included in the Brief Repeatable Neuropsychological Battery, the Brief International Cognitive Assessment for Multiple Sclerosis, and the Minimal Assessment of Cognitive Function in MS [63, 65]. The SDMT could serve as a replacement for the PASAT in clinical trials or other settings where a comprehensive assessment is needed [65].

Baseline cognitive screening with the SDMT (or alternative) when the patient is clinically stable is recommended as a minimum requirement for all adults and children aged ≥ 8 years. Baseline value could then be used to evaluate changes in therapy or following relapse and recovery cycles [63]. Clinically significant difference on the SDMT has been defined as a 4-point score change, 10% reduction in score, score change of 0.5 SDs, or use of Reliable Change Indices [63]. Annual cognitive re-assessment with the same instrument is recommended for pwMS [63]; evidence from a long-term study in patients treated with natalizumab suggests a practice effect when SDMT is performed on a monthly basis [66].

Data from longitudinal studies ranging from 1 to 3 years have shown progressive decline in cognitive functioning in pwMS, suggesting that cognition could decline over longer periods of time (10–20 years) [63]. Furthermore, correlation between EDSS progression and reduction in SDMT performance has been demonstrated [67, 64]. Patient’s education level should be considered when making decisions based on test results [68].

A meta-analysis of studies performed in healthy subjects associated regions of the frontoparietal attentional network and occipital cortex, cuneus, precuneus, and cerebellum with performing the SDMT [69]. In addition, a systematic literature review found six studies with statistically significant confirmation of an association between decreases in SDMT and brain volume loss [70]. Consequently, damage to these brain areas or evidence of brain volume loss may indicate increased likelihood of cognitive impairment occurring in such individuals and highlight the importance of early initiation of disease-modifying therapy [70]. Another meta-analysis showed significant correlations between SDMT and volume of T2 lesions (*r* =  − 0.45; *p* < 0.001) and brain atrophy (*r* =  − 0.54; *p* < 0.001) [71].

The SDMT has been found to be the most sensitive individual cognitive measure for use in MS. Its many positive features make is especially useful in clinical practice to identify at-risk pwMS [72]. Some suggest it should also be considered the measure of choice for MS trials in assessing cognitive processing speed [72].

#### Paced auditory serial addition test (PASAT)

The PASAT is a useful cognitive tool with high sensitivity to detect sustained attention and information processing speed alterations [73]. It was originally developed to assess the effects of traumatic brain injury on cognitive functioning and subsequently was shown to have clinical utility in detecting impairments in cognitive processing in patients with a wide variety of neuropsychological syndromes [74]. It is a commonly employed neuropsychological test in pwMS and has been added as a cognitive test to several widely used batteries in this setting, such as the Brief Repeatable Neuropsychological Battery (BRN-B), the Minimal Assessment of Cognitive Function in Multiple Sclerosis (MACFIMS), and the MSFC.

For the PASAT, patients have to add pairs of digits by adding each digit to the immediately preceding one.[73]. Since its original format, specialized versions of the PASAT have been developed to cater to specific populations and presentations (aurally/visually). In MS patients, the PASAT-3 is used as part of the MSFC, where each digit is presented for either 3 or 2 s.[74]. The PASAT has good internal consistency and test–retest reliability [74]. Limitations of the PASAT include practice effects that impact reliability, a predisposition to ceiling effect, the impact of inherent math ability, and test-related anxiety [75]. It is generally not used either in clinical practice or clinical trials [75].

Comprehensive examination of the psychometric qualities of the PASAT compared with SDMT revealed the SDMT to be superior to the PASAT in terms of assessing cognitive processing speed, reliability, sensitivity, practicality and cost-effectiveness [72].

### Vision

#### Low-contrast letter acuity (LCLA)

LCLA is the leading evaluation of vision loss in patients with MS [76]. It uses a Sloan low-contrast chart to measure visual dysfunction. Sloan LCLA charts show gray letters of decreasing size against a white background. Each letter correctly identified is given 1 point, for a maximum score of 70. A change of 7 points is considered clinically meaningful [76]. This test was first validated by Balcer et al. [77], in a study comparing acuity at four contrast levels in pwMS and healthy volunteers. The study demonstrated a high level of inter-rater agreement (intra-class correlation coefficient 0.86 ± 0.95) and confirmed LCLA as a reliable test of both acuity and neurological dysfunction. Subsequent research with LCLA has associated it with MRI-confirmed T2 lesions and brain atrophy [78]. Additionally, decreased LCLA scores have been correlated to retinopathy, visual evoked potentials latency, and vision-related quality of life in patients with MS [79].

LCLA has advantages over the Pelli-Robson contrast sensitivity chart which has letters of uniform size that decrease in contrast [76, 80]. LCLA charts that decrease letter size permit better assessment of impairments in low-contrast vision at different letter sizes. [76]. LCLA also has advantages over the high-contrast visual acuity (HCVA) test a measure considered a standard outcome in many ophthalmologic disorders which has proven a suboptimal measure of visual dysfunction in MS [76]. The advantages of LCLA over these other commonly used charts in MS patients mean that Sloan LCLA has proven a useful visual outcome measure in MS clinical trials [76].

### Anatomical instruments

#### Magnetic resonance imaging (MRI)

MRI is an objective measure of MS disease activity in the central nervous system, which is more common than clinical relapses by an average ratio of 10–15:1 [81]. The role of MRI in MS has developed exponentially as the technique has evolved. MRI offers by far the most sensitive technique for detecting MS lesions and has proved to be a powerful tool across the whole spectrum of MS management in the clinical setting, from diagnosis, monitoring disease activity/clinical status, and prediction of prognosis; it has also proven a useful adjunctive outcome measure in trials of disease-modifying therapies (DMTs) [82].

##### Diagnosis

MRI has become a well-established tool for diagnostic purposes and facilitates the early diagnosis of MS; it is performed after clinical examination and history taking, facilitating early disease-modifying treatment. The McDonald diagnostic criteria for MS include specific MRI requirements for the demonstration of lesion dissemination in space and time [83].

The diagnostic utility of MRI is high, with sensitivity and specificity of up to 87 and 73 percent, respectively, for the McDonald criteria requirement of dissemination in space [84]. MRI detects many more MS lesions than computed tomography (CT), and it is able to detect MS demyelinating plaques in regions that are rarely abnormal on CT [85]. Most lesions visualized by MRI correlate with pathologic lesions [85].

##### Prognosis and disease progression monitoring

A role of MRI in monitoring relapsing MS disease progression has evolved with use, and the evolution will continue with the development of new techniques that increase the sensitivity of the instrument.

In an early meta-analysis by Kappos et al. [86], the standard deviation of the number of gadolinium-enhancing (Gd+) lesions predicted relapse rates in the next year. However, the researchers found no statistically significant association between Gd+ lesion count at study initiation and EDSS score at 1 or 2 years [86], A subsequent meta-analysis suggested that MRI findings could serve as an alternative endpoint to relapses in clinical trials of MS [87].

New lesion formation is the best MRI biomarker of active inflammation in relapsing MS and predicts poor outcome during interferon treatment [81]. A study of patients with early-onset clinically isolated syndrome (*n* = 178) provided evidence that baseline Gd+ and spinal cord lesions are independently associated with secondary progressive MS at 15 years and showed a consistent association with EDSS [88]. Based on these findings, the authors concluded that spinal cord lesions observed on MRI anticipate poor outcomes, disease progression, and relapse-onset MS [88]. Findings from MRIs may, therefore, be useful for discussing long-term prognosis and treatment plans with patients [88]. Despite their diagnostic utility, MRI lesion scans are difficult to quantify and pathology must be interpreted [81].

In addition to imaging lesions, MRI can be used for volumetric analysis of both whole and regional brain atrophy, which anticipates worsening ambulatory and cognitive function in pwMS [89]. Data from a 3-year prospective observational study in an MS population (*n* = 1052) showed a significantly increased prevalence of cognitive impairment in patients with brain atrophy and high lesion volume. Patients with brain parenchymal fraction < 0.85 and T2 lesion volume > 3.5 mL were more likely to have cognitive impairment compared with patients with brain parenchymal fraction > 0.85 and T2 lesion volume < 3.5 mL (odds ratio 6.5; 95% CI 4.4–9.5) [90]. In an MRI study in 61 patients with relapsing–remitting MS, those with cognitive impairment had significant differences in MRI-detected markers of brain atrophy [91]. Volumetric analysis has also correlated whole-brain atrophy with dysarthria (*r* = 0.46; *P* < 0.001) [92].

Data from observational studies have confirmed that thalamic atrophy is highly predictive of cognitive decline and neurodegenerative processes [93–95]. A recent study in patients with secondary progressive MS provided evidence that atrophy of the corpus callosum also predicts cognitive decline, with detriment to employment [96].

##### Assessing treatment response in clinical trials

Most often, clinical trials of pharmacologic treatments include MRI findings as a secondary outcome measure, using changes in the amount and size of T2-hyperintense and contrast-enhanced T1-hypointense lesions [97]. One meta-analysis of MS intervention trials assessed the effect of treatment on lesion burden. The analysis of 31 studies revealed that treatment effects on MRI lesions over 6–9 months can be predictive of relapses over 12–24 months. Furthermore, new or enlarging T2-hyperintense lesions and contrast-enhanced T1-hypointense lesions were associated with the number of relapses and MRI was subsequently suggested as a primary outcome measure for treatment trials [87].

#### Optical coherence tomography (OCT)

OCT is a simple office-based measure that uses near-infrared light for rapid cross-sectional imaging of the back of the eye [98]. Visualization of retinal tissue is of specific interest in MS because axons comprise a tissue layer in the retina, the retinal nerve fiber layer (RNFL) [99]. Moreover, this is a unique location within the central nervous system to assess axonal volume exclusively as the ganglion cell axons are unmyelinated (therefore, the volume change of myelin is a non-factor). OCT allows visualization of neurodegenerative changes in the retina and has the potential to be a useful tool for measuring the impact of treatment on neurodegeneration in pwMS [100]. Advantages of OCT over MRI include accessibility and technical ease [100]. The OCT can be performed at lower cost and with a shorter image duration.

Time domain was the first OCT technique used in pwMS [101]. Spectral OCT has become the preferred technique because it facilitates visualization of additional retinal layers and quantification of their thicknesses [98]. RNFL thickness indicates axonal injury independent of myelin sheath presence or thickness [102].

A meta-analysis of studies on time domain OCT and MS published through May 2010 included 32 studies [99]. When compared with healthy controls, RNFL loss was− 7.08 (95% CI − 8.65 to − 5.52) μm in pwMS with no history of optic neuritis and − 20.38 (− 22.86 to − 17.91) μm in pwMS with associated optic neuritis. An updated meta-analysis for data published on spectral OCT and MS through April 2016 included 40 studies [101]. Comparing eyes of pwMS with and without associated optic neuritis, the inner nuclear layer was thinner in individuals with optic neuritis-associated MS than those without. The RNFL layer was thinner in both populations compared with the RNFL thickness in healthy controls. Atrophy of the ganglion cell layer and inner plexiform layer was greater in all pwMS (with and without associated optic neuritis) than in healthy controls and was greater in individuals with MS associated with optic neuritis than in those without.

For pwMS from a single center who had OCT results available, a lower total macular volume at baseline was associated with a higher 10-year EDSS score [103]. This association was stronger in the lowest one-third of the baseline macular volume score and for those individuals with relapsing–remitting MS [103]. For each 1-year increase in the duration of disease, there was an associated decrease of 0.2% in the superficial vascular plexus; and overall, lower density was associated with higher EDSS scores [104]. In addition, optic nerve diameter and RNFL thickness were significantly lower in individuals with an EDSS score > 2 than in those with an EDSS score ≤ 2 [105]. Moreover, researchers have shown correlations between diminished RNFL thickness on OCT and MRI volumetric degeneration of the corpus callosum [106] and brain parenchymal fraction and cerebrospinal fluid (CSF) volume [107] and correlation between rates of ganglion cell + inner plexiform layer and whole brain atrophy [108]. These findings provide evidence that ocular damage occurs simultaneously to brain atrophy in pwMS.

Some studies indicate that OCT may be less sensitive than visual-evoked potentials (VEP) for detecting lesions of the visual pathway in early relapsing–remitting MS patients [109]. However, the two techniques may be useful when used complementarily since VEP may be a better tool for detecting early demyelinating lesions whereas OCT may be a better tool for monitoring axonal loss and neurodegeneration.

### Biological instruments

Specific biological markers that can assist the clinician in monitoring specific MS treatments, such as natalizumab and interferon beta, have been reviewed elsewhere [110, 111].

Biological biomarkers under investigation for prognostic use in MS include oligoclonal bands (OCBs) and chitinase-3-like protein 1 (CHI3L1) [112]. Levels of immunoglobulin G (IgG) OCBs and neurofilaments in CSF have been shown to anticipate conversion of demyelination symptoms to clinically isolated syndrome [113]. Prospective analysis of MRI data in the Swedish Multiple Sclerosis Registry-associated OCBs with whole-brain atrophy and decreased white matter [114]. In addition, retrospective and prospective studies have shown: numerical differences in disease severity based on the number of IgG OCBs [115], significantly higher levels of disease activity in patients with versus without IgM OCBs [116], and aggressive disease development in patients with IgM OCBs [117]. The glycoprotein CHI3L1 has also been shown to be predictive of long-term impairment and CDMS in patients whose first demyelinating event was optic neuritis [118] and in patients with monophasic neurological symptoms [119]. However, these markers may not be useful in routine clinical practice.

One of the more promising biomarkers for monitoring disease progression in MS is neurofilament light chain (NfL). Neurofilaments—cytoskeletal components of neurons—are abundant in axons and include heavy, medium, and light chain filaments [120]. In patients with axonal damage, neurofilament concentrations increase to abnormal levels [121]. Increased NfL concentrations in CSF have been observed in individuals with MS compared with healthy controls [122, 123]. Moreover, elevated concentrations of NfL in CSF correlate with measures of MS disease progression [124] and treatment effects [123, 125].

Advances in the technological assessment of NfL concentrations have facilitated the measurement of NfL in the serum (sNfL). Recent evidence suggests that sNfL has the potential to be useful in the monitoring of response to disease-modifying therapy in individuals with MS [124, 126–128]. Validation of a reliable assay coupled with further clarification of the relationship between sNfL and disease progression or treatment monitoring may position this biological marker as a routine assessment of MS activity.

## Summary

Since the introduction of the EDSS in 1983, numerous tests and instruments have been developed for the assessment of patient function and progression of MS. These instruments have enhanced the ability of the clinician to identify changes in pwMS that otherwise might be missed in a purely clinical assessment. Early identification of patient conditions that require symptomatic interventions or optimization of disease-modifying therapies may result in better outcomes. Moreover, these instruments are objective measurements of the disease evolution. Rather than evaluate and comment on all available instruments, we have focused on those that are most useful in clinical practice based on ease of administration, objective quantitative results, and applicability in clinical practice.

As complexity and heterogeneity are hallmarks of MS, the diagnosis and management of the disease require a combination of clinical scales, imaging techniques and laboratory findings to monitor and quantify symptomatic complications as well as underlying pathological events. Each technique has advantages and deficiencies and none is an ideal outcome measure; thus, a combinatory approach of both clinical rating scales and imaging techniques can help to provide a more holistic picture of disease progression. Rating scales targeted at specific variables (e.g. motor strength, spasticity, walking ability) can provide information about the symptomatic impact of the disease to the individual patient while MRI is able to provide information about the underlying pathology as well as essential prognostic detail. For diagnostic purposes, MRI evidence plays a supportive role in what is ultimately a clinical diagnosis of MS, since MRI abnormalities can be associated with other diseases and non-specific MRI lesions are also common in the general population. CSF analysis of oligoclonal bands, visual evoked potentials, and OCT can all be used to support diagnosis in patients with typical presentation who have insufficient clinical and MRI evidence to confirm the diagnosis [83].

In addition to the instruments discussed in this review, new tools continue to be validated for use in pwMS. Electronic self-assessment instruments provide innovative opportunities for patient engagement in the clinical setting. The Performance Speed Test (PST) employs tablet software for patient-administered screening of cognitive dysfunction [129], and the MS Performance Test (MSPT) is tablet-based with modules for cognition and motor function [130, 131]. The Multiple Sclerosis Partners Advancing Technology and Health Solutions (MS PATHS) initiative, a learning health system being developed by institutions in 10 countries in collaboration with Biogen, is using the MSPT to standardize information related to patient care in MS clinical practices [132].

Wearable biosensors will also open new avenues for collecting patient data on ambulation, balance, and physical activity or function. New technologies will add real-life details that will allow clinicians to better understand disease progression in their patients and personalize treatment. Ultimately, though, these technologies cannot take the place of clinical evaluations by trained health care providers using the validated modalities discussed in this review. In addition to providing standardized methodology to record patient history, these modalities are well understood by the MS community. Essential work over the decades since EDSS was introduced continues to improve our ability to treat this debilitating disease.

## Data Availability

Not applicable.
